# Blood Cardioplegia Induction, Perfusion Storage and Graft Dysfunction in Cardiac Xenotransplantation

**DOI:** 10.3389/fimmu.2021.667093

**Published:** 2021-06-09

**Authors:** Corbin E. Goerlich, Bartley Griffith, Avneesh K. Singh, Mohamed Abdullah, Shreya Singireddy, Irina Kolesnik, Billeta Lewis, Faith Sentz, Ivan Tatarov, Alena Hershfeld, Tianshu Zhang, Erik Strauss, Patrick Odonkor, Brittney Williams, Ali Tabatabai, Adnan Bhutta, David Ayares, David J. Kaczorowski, Muhammad M. Mohiuddin

**Affiliations:** ^1^ Department of Surgery, The University of Maryland School of Medicine, Baltimore, MD, United States; ^2^ Department of Surgery, Johns Hopkins School of Medicine, Baltimore, MD, United States; ^3^ Department of Cardiothoracic Surgery, Cairo University, Cairo, Egypt; ^4^ Department of Medicine, Division of Pulmonary and Critical Care Medicine, University of Maryland School of Medicine, Baltimore, MD, United States; ^5^ Department of Pediatrics, The University of Maryland School of Medicine, Baltimore, MD, United States; ^6^ Revivicor, Inc., Blacksburg, VA, United States

**Keywords:** xenotransplantation, graft dysfunction, cardiac xenotransplantation, cardiac preservation, heart transplant, heart failure, ventricular assist device (VAD)

## Abstract

**Background:**

Perioperative cardiac xenograft dysfunction (PCXD) describes a rapidly developing loss of cardiac function after xenotransplantation. PCXD occurs despite genetic modifications to increase compatibility of the heart. We report on the incidence of PCXD using static preservation in ice slush following crystalloid or blood-based cardioplegia versus continuous cold perfusion with XVIVO^©^ heart solution (XHS) based cardioplegia.

**Methods:**

Baboons were weight matched to genetically engineered swine heart donors. Cardioplegia volume was 30 cc/kg by donor weight, with del Nido cardioplegia and the addition of 25% by volume of donor whole blood. Continuous perfusion was performed using an XVIVO ^©^ Perfusion system with XHS to which baboon RBCs were added.

**Results:**

PCXD was observed in 5/8 that were preserved with crystalloid cardioplegia followed by traditional cold, static storage on ice. By comparison, when blood cardioplegia was used followed by cold, static storage, PCXD occurred in 1/3 hearts and only in 1/5 hearts that were induced with XHS blood cardioplegia followed by continuous perfusion. Survival averaged 17 hours in those with traditional preservation and storage, followed by 11.47 days and 15.03 days using blood cardioplegia and XHS+continuous preservation, respectively. Traditional preservation resulted in more inotropic support and higher average peak serum lactate 14.3±1.7 mmol/L compared to blood cardioplegia 3.6±3.0 mmol/L and continuous perfusion 3.5±1.5 mmol/L.

**Conclusion:**

Blood cardioplegia induction, alone or followed by XHS perfusion storage, reduced the incidence of PCXD and improved graft function and survival, relative to traditional crystalloid cardioplegia-slush storage alone.

**Graphical Abstract:**
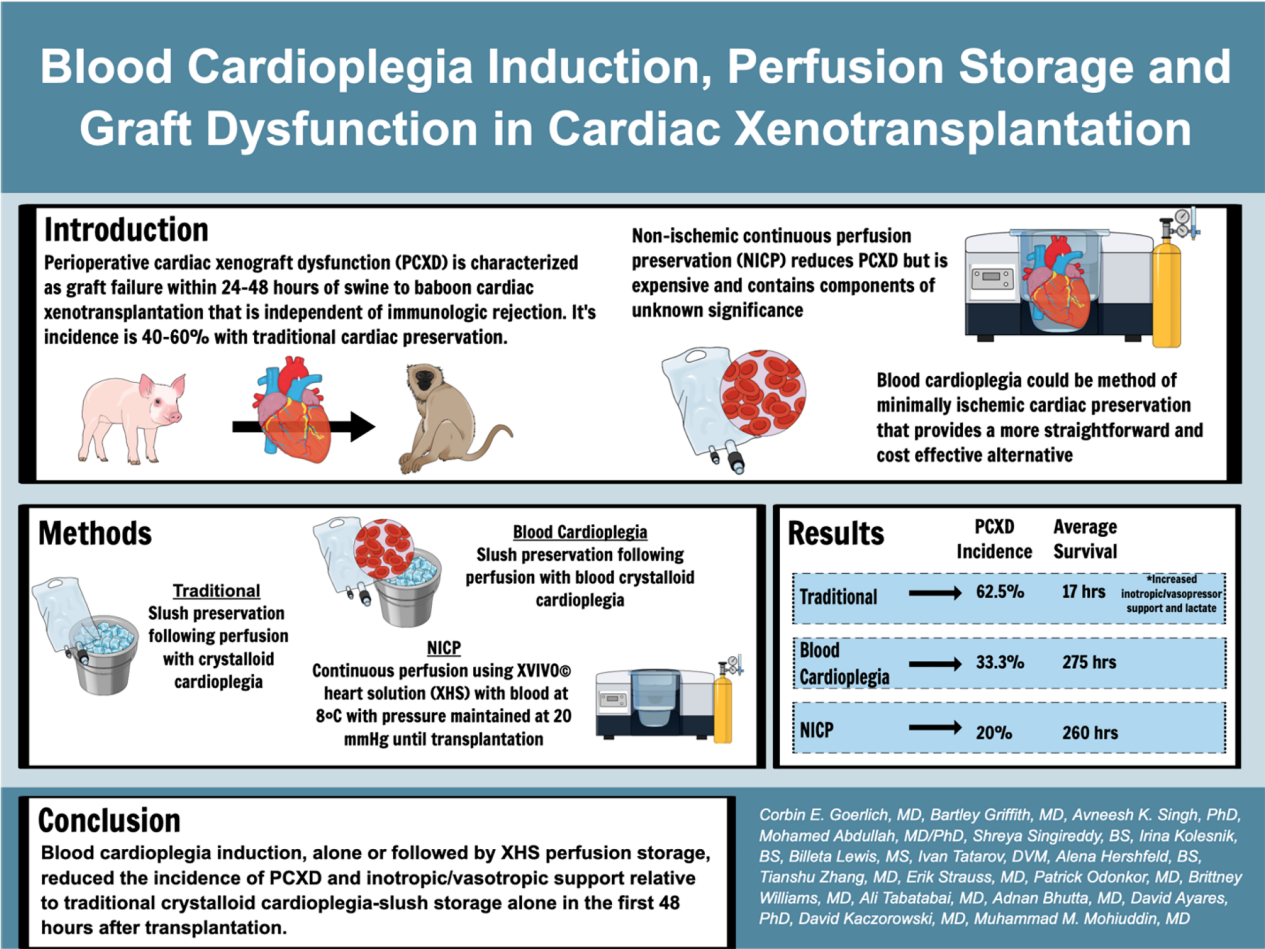


## Introduction

Heart transplantation is the optimal treatment of end-stage heart failure after failure of maximal medical therapy. However, the shortage of donor hearts restricts its utilization. At any given time, 3,000-4,000 people are waiting for a heart transplant in the United States (1). Xenotransplantation of genetically engineered porcine hearts for human transplantation has been proposed as an alternative approach. However, perioperative cardiac xenograft dysfunction (PCXD) has been observed in 40-60% of orthotopic cardiac xenotransplants and is considered a significant barrier to translational use in humans (2). PCXD is characterized as graft failure within 24-48 hours of transplantation that is independent of immune organ rejection. It has been considered to be associated with ischemia-reperfusion (I/R) injury and a systemic inflammatory response to xenotransplantation (3–6). Mitigating PCXD and inflammation has resulted in up to 6 months of survival in pig-to-baboon cardiac xenotransplants, using a non-ischemic continuous perfusion preservation technique (NICP) and anti-inflammatory agents, among other adjuncts (7). NICP prevents I/R injury and has been shown to ease removal from cardiopulmonary bypass and reduce the need for catecholamine support (3). However, NICP is expensive and the components of the oxygenated perfusate are complex with components of unknown significance (8).

In allotransplantation, traditional static preservation is well tolerated and has been used since the inception of heart transplantation, resulting in primary graft dysfunction (PGD) in 8-10% (9, 10). For reasons that are not entirely clear, traditional preservation techniques are not sufficient to prevent PGD during cardiac xenotransplantation. In contrast to traditional static preservation (i.e., slush), NICP strategy utilizes a hyperoncotic cardioplegic perfusate containing packed red blood cells, human serum albumin (HSA), dextran 40, inotropes, hormones, antibiotics, and cocaine to minimize ischemia-reperfusion phenomena and prevent myocardial edema (8).

We aimed to determine whether blood cardioplegia with freshly oxygenated donor blood before donor cardiectomy, followed by storage on ice, could similarly prevent PCXD and increase cardiac xenotransplantation recipient survival compared to NICP. This method of minimally ischemic cardiac preservation provides a potentially more straightforward and cost-effective alternative to the NICP preservation technique.

## Methods

### Animal Model

Genetically engineered German landrace pigs were of either sex, 15-30 kg, of non-AB (O) blood type, and cytomegalovirus (CMV)-negative. Donor swine were provided by Revivicor, Inc. and were all α-1,3-galactosyltransferase gene knock-out (GTKO) with additional human transgene expression (**Table 2**), depending on organ availability. The porcine hearts were transplanted into similarly sized *Papio albus* baboons of either sex, screened for the absence of select pathogens. All animals were used in compliance with “Guide for the Care and Use of Laboratory Animals” recommended by the National Institutes of Health and the University of Maryland Institutional Animal Care and Use Committee (IACUC).

### Procedures

#### Traditional Cardioplegia Induction, With Static Preservation

After ligating the superior and inferior vena cava, either Custodial crystalloid cardioplegia or Del Nido solution without blood was administered through the ascending aorta at a volume of 30cc/kg. The heart was then procured and stored on ice (slush) until transplantation.

#### Crystalloid Blood Cardioplegia, With Static Preservation

Blood cardioplegia induction included del Nido cardioplegia solution (containing Plasma-Lyte A, Mannitol 20%, MgSO_4_ 50%, NaHCO_3_ 8.4%, KCl, Lidocaine 1%) with fresh donor blood collected just prior to administering cardioplegia at 25% of total cardioplegia volume (11). Just as traditional means, the heart was then stored on ice until transplantation.

#### Non-Ischemic Continuous Perfusion (NICP)

Blood cardioplegia induction included XHS cardioplegia with fresh donor blood collected just prior to administering cardioplegia at 25% of total cardioplegia volume (similar to above). After procuring the swine heart, it was connected to the XVIVO^©^ perfusion system (XVIVO^©^ Perfusion, Gothenburg, Sweden) and continuously perfused with oxygenated XHS at 8°C with pressure maintained at 20 mmHg at a physiological pH (7.2-7.6) until transplantation (8). An XVIVO^©^ dual lumen cannula (central inflow, axially oriented outflow and quick connects for machine circuit connection) was placed in the transected ascending aorta, with the tip of the cannula in the root, just above the aortic valve. Care was taken to prevent aortic valve incompetence as a result of cannula placement. The mitral valve was made incompetent by transvalvular placement of silicon tubing and sutured to the left atrium, in order to prevent LV distention.

### Recipient Immunosuppression

An anti-CD40 monoclonal antibody (mAb)-based immunosuppression regimen was used for all recipients of this study. This has been extensively described elsewhere (12). Briefly, induction was performed with anti-CD20 mAb, thymoglobulin, cobra venom factor, and anti-CD40 mAb, and maintenance consistent of anti-CD40 mAb, mycophenolate mofetil (MMF or Cellcept), and steroids.

### Resuscitation

Standard resuscitation with crystalloid, colloid, inotropes, pressers and blood products was performed in order to maintain MAP >60, Hgb >8.0, adequate oxygenation/ventilation, acid/base homeostasis and adequate organ perfusion in the immediate postoperative period. Critical Care nurses and physicians provided ICU level monitoring and management for the first 48-72 hours postoperatively.

### Anesthesia

Details of anesthesia induction and maintenance and rationale for agent selection has been detailed previously (13). Briefly, the donor is sedated with 10mg/kg of Ketamine and 2mg/kg of xylazine intramuscularly and transferred to the operating room. The recipient is similarly sedated with 10mg/kg of intravascular ketamine through a tunneled central line and brought to the operating room. Both donor and recipients are intubated and induced with isofluorene (1-1.5%) for a goal minimal alveolar concentration of 1.0-1.2. Analgesia and paralysis are maintained with fentanyl and succinylcholine, respectively.

### Euthanasia

Recipients were euthanized upon determining that standard resuscitation would not lead to meaningful recovery of xenograft function or recipient physiologic derangements such as acidosis or hypoxemia. Additional euthanasia criteria were for intractable arrythmias.

### Objective Quantification of Support and PCXD Postoperatively

In order to objectively quantify the amount of support required postoperatively, inotrope and vasopressor support were quantified on a 1-5 scale as depicted in **Table 1**. Support was quantified at the time of weaning cardiopulmonary bypass (CPB), 1 hour, 12 hours and 24 hours after weaning CPB and the summation of each time point was tabulated. PCXD was defined as graft failure/intractable arrythmia leading to euthanasia prior to 48 hours after transplantation.

**Table 1:** Objective Quantification of Support Postoperatively-ionotropic and vasopressor support required in the first 24 hours after transplantation was quantified by the following 1-5 scale, with additive scores for each drug used for 0, 1, 12 and 24 hour time points.

Support Scale	1	2	3	4	5
Inotropes:					
Epinephrine (mcg/kg/min)	0.01-0.05	0.06-0.01	0.11-0.15	0.16-0.20	> 0.20
Dobutamine (mcg/kg/min)	2.5-6.9	7.0-11.4	11.5-16.9	16.0-20.4	> 20.4
Milrinone (mcg/kg/min)	0.125-0.24	0.250-0.374	0.375-0.49	0.5-0.624	> 0.624
Vasopressors:					
Norepinephrine (mcg/kg/min)	0.01-0.1	0.11-0.2	0.21-0.3	0.31-0.4	> 0.4
Phenylephrine (mcg/kg/min)	0.1-0.9	1.0-1.9	2.0-2.9	3.0-4.0	> 4.0
Vasopressin (units/min)	0.01	0.02	0.03	0.04	> 0.04

### Statistical Analysis

All statistical analyses, including Kaplan Meyer curves, ANOVA and t-tests were performed on Graphpad Prism version 9 (San Diego, CA).

## Results

**Figure 1:**
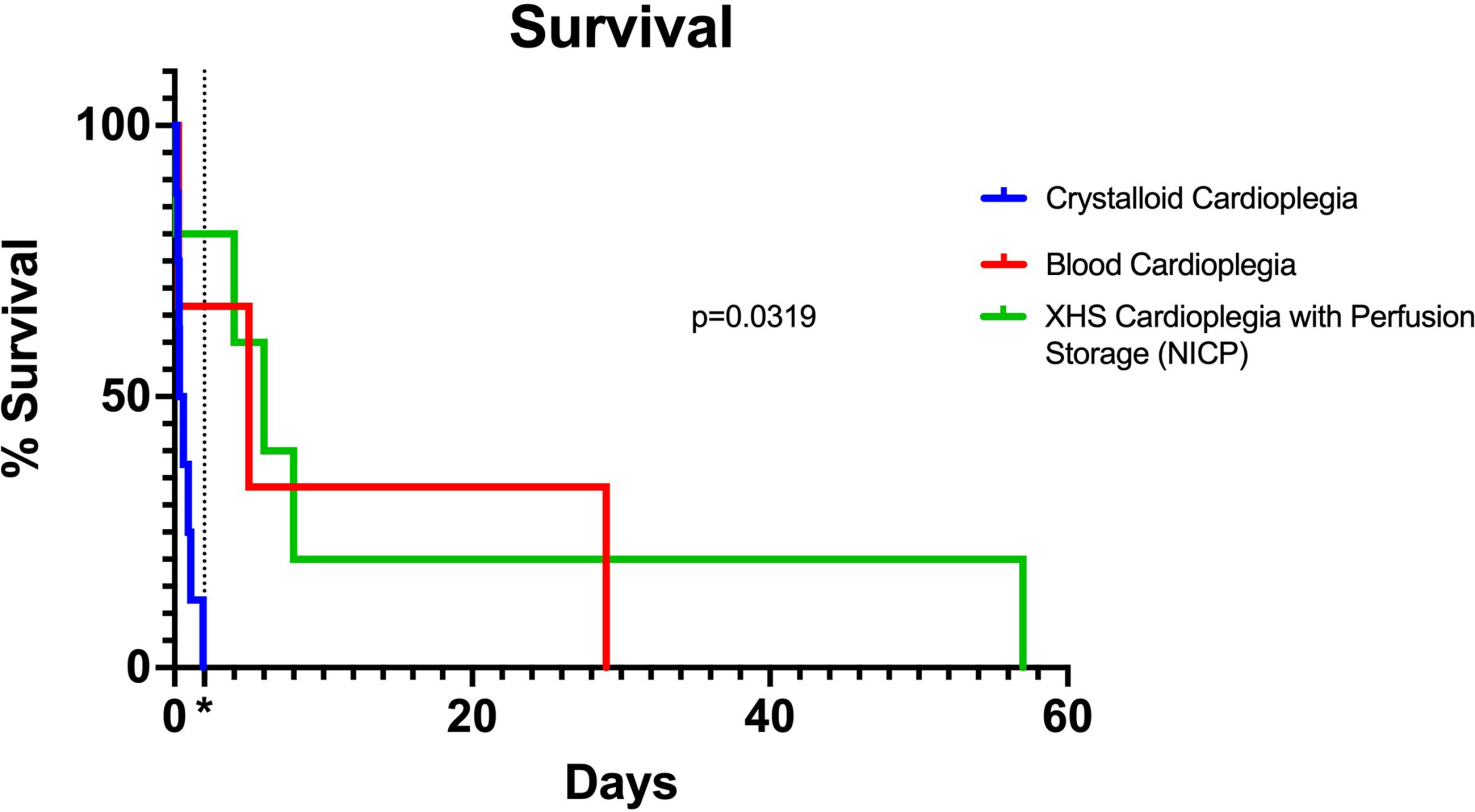
Kaplan Meier curve comparing traditional, blood cardioplegia and NICP preservation methods. P-value calculated by Log-rank (Mantel-Cox) test. *=48 hours (2 days) after transplantation, indicating survival beyond PCXD.

A summary of the results of the study are shown in **Table 2**. There were no significant differences between donor and recipient weights, cross clamp times, total ischemia times and cardiopulmonary bypass times between traditional crystalloid cardioplegia, blood cardioplegia and NICP groups (**Table 3**). Survival for recipients with donor xenografts that underwent traditional cardioplegia induction and storage in slush ranged from 3 to 26 hours, with an average of 17 hours. Blood cardioplegia induction and storage in slush resulted in a drastic improvement in xenograft survival with an average of 275 hours (11.45 days), with a maximum survival of 29 days (**Figure 1**) (p=0.0319). Similarly, NICP extended survival time to an average of 360.6 hours (15.03 days), with a maximum survival of 57 days (**Figure 1**). PCXD (i.e., graft failure, without histiologic/immunologic evidence of rejection, within 48 hours of xenotransplant) was overcome in 2 out of 3 with blood cardioplegia/slush storage and 4 out of 5 NICP storage, respectively.

**Table 2:** Comparison of Traditional, Blood Cardioplegia and NICP preservation strategies.

Preservation Type	Crystalloid Induction with Slush Storage							
Genetics	GTKO.B4KO.hCD46.hTBM.hEPCR.hCD47.hHO1.hVWF	GTKO.hCD46.hTBM.hCD47.hEPCR.hHO1	GTKO.hCD46.hTBM.hCD47.hEPCR.hHO1	GTKO.hCD46	GTKO.B4KO.hCD46.hHLAE	GTKO.CMAHKO.hCD46.hCD47.hTFPI	GTKO.CMAHKO.hCD46.hEPCR.hDAF.hTBM.hHO1	GTKO.hCD46.hTBM
**Survival**	26 hours	3 hours	22 hours	14 hours	7 hours	5 hours	8 hours	46 hours
**Donor weight (kg)**	21	10	23	13	9	20	20	29
**Recipient weight (kg)**	25	10	21	13	10	18	20	30
**Cross clamp time (minutes)**	95	96	113	69	65	185	55	74
Total ischemia time*	95	130	–	–	142	> 185	46	69
**CPB time (minutes)**	131	164	122	118	147	107	163	148
**Time on XVIVO (min)**	–	–	–	–	–	–	–	–
**Preservation Type**	**Blood Cardioplegia Induction with Slush Storage**			**Non-Ischemic Continuous Preservation**				
**Genetics**	GTKO.hCD46.hTBM	GTKO.hCD46.hTBM	GTKO.hCD46.hTBM	TKO.hCD46.hDAF	TKO only	GTKO.hCD46.hTBM	GTKO.hCD46.hTBM	GTKO.hCD46.hTBM
**Survival**	6 hours	5 days	29 days	6 days	8 days	57 days	3 hours**	4 days
**Donor weight (kg)**	12	20	21	18	24	21	16	21
**Recipient weight (kg)**	13	17	21	22	23	22	16	30
**Cross clamp time (minutes)**	142	50	49	63	31	53	54	–
Total ischemia time*	–	55	95	210	159	156	137	168
**CPB time (minutes)**	152	92	102	132	91	98	116	117
**Time on XVIVO (min)**	–	–	–	124	107	98	85	101

*total ischemia time includes time in XVIVO^©^ perfusion box for NICP OHTx. **, survival limited due to sudden v-fib arrest upon emergence from anesthesia. –, missing datapoints. n/a, not applicable; GTKO, α1,3-galactosyltransferase knockout; TKO, (triple knockout; GTKO+B4GalKO+CMAHKO); B4GalKO, β1,4-N-acetylgalactosyltransferase knockout; CMAHKO, CMP-N-acetylneuraminic acid hydroxylase knockout; TBM, thrombomodulin; EPCR, endothelial protein C receptor; DAF, decay accelerating factor; HO1, hemeoxygenase; GHRKO, growth hormone receptor knockout; V-fib, ventricular fibrillation; AMR, antibody-mediated rejection.

**Table 3:** Mean survival, descriptive statistics between traditional, blood cardioplegia and NICP.

Preservation Type	Traditional (n=8)	Blood Cardioplegia (n=3)	NICP (n=5)	p-value
**Survival- hours (days)**	16 (0.68) ± 15	274 (11.42) ± 370	361 (15.03) ± 567	p=0.0139
**Cross clamp time (minutes)**	94 ± 41	80 ± 53	50 ± 14	ns
Total ischemia time (minutes)*	111 ± 51	75 ± 28	166 ± 27	ns
**CPB time (minutes)**	138 ± 21	115 ± 32	111 ± 16	ns
**% Extubated**	0.00%	66.70%	100.00%	n/a
**% Surpassed 48-hour survival**	0.00%	66.70%	80.00%	n/a
**% PCXD**	62.5%	33.3%	20.0%	n/a

Mean=mean ± standard deviation. CPB, cardiopulmonary bypass; PCXD, perioperative cardiac xenograft dysfunction. *total ischemia time includes time in XVIVO box for NICP OHTx and thus only traditional vs. blood cardioplegia means are compared. Otherwise, ANOVA is used to compare all 3 groups with each other for each variable. ns, not significant; n/a, not applicable.

**Figure 2:**
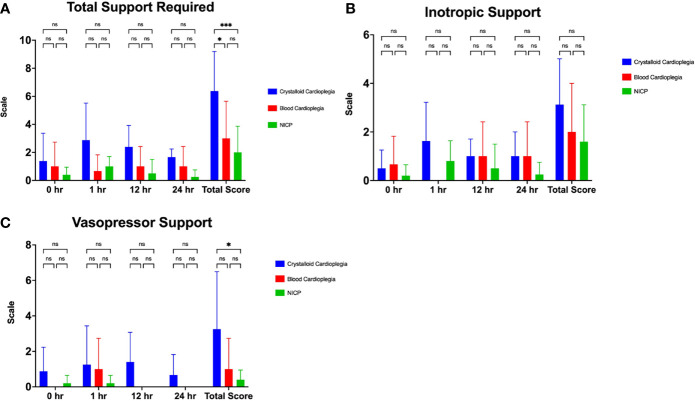
**(A)** Total support required in the first 24 hours after transplantation in crystalloid cardioplegia, blood cardioplegia and NICP groups, based on objective quantification from **Table 1**. **(B, C)** Inotropic and vasopressor scores in the first 24 hours after transplantation between groups. Total scores were calculated as the summation of all scores for each time point. *p=<0.05, **p<0.005, ***p<0.0005. ns, not significant.

NICP and blood cardioplegia resulted in significantly less support (**Figure 2**). While the average support score for traditional preservation was 6.4, the scores for blood cardioplegia induction and NICP were 3.0 and 2.0, respectively (p= 0.0294 and 0.0005, respectively) (**Figure 2A**). When support scores are further stratified by both inotropy and vasopressor requirements, there was a trend toward less inotropy requirements in blood cardioplegia and NICP preservation strategies, compared to traditional crystalloid cardioplegia but it did not reach statistical significance (**Figure 2B**). There was a statistically significant reduction in vasopressor requirement in NICP compared to blood cardioplegia and traditional preservation (p=0.0117) (**Figure 2C**).

**Figure 3:**
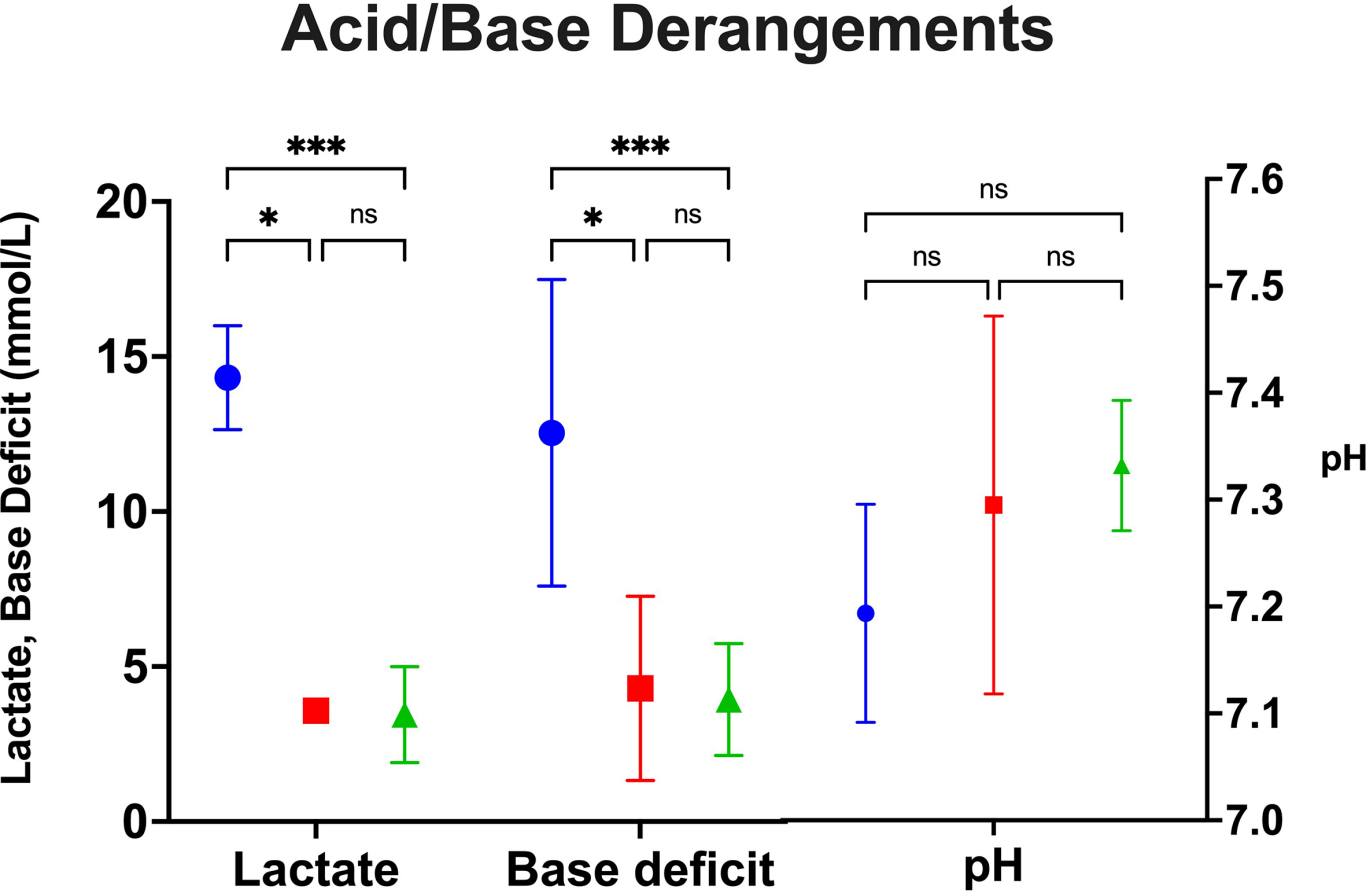
Peak acid/base derangements as measured by lactate, base deficit and pH between groups. *p=<0.05, **p<0.005, ***p<0.0005. Blue=Crystalloid Cardioplegia, Red=Blood Cardioplegia, Green=NICP. ns, not significant.

Recipients with traditionally preserved hearts average higher maximum lactate (14.3 mmol/L) and base deficit (12.5 mmol/L) compared to blood cardioplegia and NICP (**Figure 3**). Recipients that underwent OHTx utilizing blood cardioplegia induction followed by slush preservation strategies resulted in an average maximum lactate and base deficit levels of 3.6 mmol/L and 4.3 mmol/L, respectively (p=0.0187, p=0.0123, respectively compared to crystalloid cardioplegia). NICP resulted in similar average maximum lactate and base deficit levels of 3.5 mmol/L and 3.9 mmol/L, respectively (p=0.0001, p=0.0005, respectively). While differences in pH were not statistically significant, the differences are clinically significant.

## Discussion

This study confirms that traditional static preservation using cold cardioplegic solution followed by storage on ice results in poor xenograft survival. The observed benefit in this study with the use of alternative approaches to cardiac xenograft preservation while not conclusive, is certainly hypothesis-generating. The issue may lie in cardiac xenografts’ increased response to ischemia-reperfusion injury, as demonstrated by improved outcomes in this study by employing progressive levels of cardiac preservation with oxygenated blood. Notably, Längin, et al. has demonstrated significantly depressed systolic function leading to early graft failure when traditional cardioplegia was employed, compared to NICP (6). In this study, the potential merit of blood cardioplegia for induction and preservation of cardiac xenografts to overcome PCXD as a less costly and cumbersome alternative to NICP is discussed.

There is literature to support our observations. It is known that during solid organ procurement, preservation and subsequent reperfusion result in oxygen radical formation, edema, microvascular injury, and compromised microcapillary circulation (14–17). The underlying mechanism is likely two-fold: a continued ischemic phenomenon, despite reperfusion, and a paradoxical progression of damage due to the reperfusion of ischemic areas (i.e., ischemia-reperfusion injury). There are definitive multifocal, patchy ischemic areas that result upon reperfusion of ischemic striated muscle due to either constriction of afferent arterioles, obstruction of the capillaries themselves or interstitial edema (18). Secondly, reperfusion injury occurs upon restoration of flow in ischemic areas due to oxygen free radical formation from NADPH and xanthine oxidase, causing endothelial cell damage. Most relevant to xenotransplantation, oxidative stress also causes leukocyte adherence to endothelial cells, activation and further endothelial damage (19). These mechanisms of graft injury are largely augmented by recipient inflammation at the graft endothelium. Since immunological insults of much higher intensity are seen in cross-species transplantation, the differences seen in PGD in allotransplantation versus xenotransplantation can be explained by augmented ischemia-reperfusion injury by inflammation (16). Perhaps by minimizing ischemia using either blood cardioplegia induction or NICP, downstream effects of ischemia reperfusion are reduced.

However, other components of NICP perfusate (i.e., that minimize ischemia) should not be discounted. They further support the likely mechanisms of primary graft failure in cardiac xenotransplantation or PCXD. Other than oxygenated red blood cells, the primary components of NICP perfusate are human serum albumin (HSA) and dextran 40 (8). Serum albumin serves to increase the oncotic pressure intravascularly, abrogating some of the microcapillary dysfunction, while dextran 40 protects the endothelial lining from leukocyte interaction (20). To what extent each component contributes to mitigating further damage by ischemia-reperfusion is mostly unknown. However, incorporating HSA and dextran 40 into cardioplegia solution with fresh donor red blood cells should be the next step in elucidating these mechanisms. It may be that combining HSA and dextran 40 with blood cardioplegia induction strategies is equally effective as NICP, but this has yet to be studied. Moreover, it was observed that there was significantly less vasopressor requirement in recipients of xenografts that received NICP, suggesting vasoplegia after transplantation is reduced. This observation should be interpreted with caution, as the sample size is limited, but it does provide potential evidence that NICP mitigates inflammation-induced vasoplegia not seen by other preservation strategies.

Blood cardioplegia efficacy in overcoming PCXD was quite remarkable in and of itself and this study may underrepresent its benefit. The one recipient in the group induced with blood cardioplegia, followed by slush storage that did not overcome PCXD had a notably higher cross-clamp time (142 minutes) and cardiopulmonary bypass time (152 minutes) than the averages of our institution and was a more technically difficult transplant. This may have conferred a worse outcome than would have otherwise been exhibited with standard operating times. Necropsy demonstrated an unusually large amount of multifocal contraction band necrosis, characteristic of ischemia-reperfusion injury.

A notable limitation to this study is in the different genetic backgrounds of the donor xenografts between groups. This could lead the reader to believe that these differences could influence xenograft function, lead to early rejection and influence survival. Indeed, genetic differences could (and may) have influenced overall survival, however, this was only a tertiary endpoint of this study. The primary endpoint of this study was the incidence of transplants that overcame PCXD, and secondary endpoints included the amounts of support and metabolic derangements that occurred within the first 24 hours after transplantation. To that end, all xenografts contained at least GTKO and expression of one complement regulatory and thromboregulatory protein (which are known principle components of genetically engineered grafts shown to prevent hyperacute rejection in cardiac xenotransplantation) (21, 22). Thus, genetic differences of the xenografts in this study are likely negligible. One exception is in the NICP group, where a xenograft lacked complement regulatory proteins and thromboregulatory proteins (“TKO only” xenograft). However, this also underscores the point regarding genetic differences in that this graft surpassed PCXD and had minimal metabolic derangements after transplantation despite the limited genetic modifications of this xenograft.

Lastly, it should be noted that information gained from this study may be applicable to allotransplantation. Like xenotransplantation, PGD is the leading cause of mortality in patients undergoing heart transplantation (9). Despite advances in allocation methods, cardiopulmonary bypass, and postoperative care, the incidence is still around 8-10% (9). Indeed, NICP, initially developed for xenotransplantation, has since gained traction in allotransplantation in a Phase II clinical trial (10). While blood cardioplegia in the context of coronary artery bypass grafting (CABG) and aortic root repair has been trialed, results are mixed and have not been tested in the context of allotransplantation (23).

## Conclusion

We have shown that both blood cardioplegia induction and NICP increase survival in cardiac xenotransplantation compared to traditional static preservation. Blood cardioplegia induction and NICP show similar prevention of metabolic derangements and need for support postoperatively, but NICP shows greater incidence of progression past PCXD compared to blood cardioplegia. However, with the simple addition of fresh donor blood to traditional cardioplegia, still shows some merit as demonstrated by a significant improvement in both progression of PCXD and survival. Likely, a combination of the properties of red blood cell containing perfusate used in NICP (namely, HSA and dextran 40) with freshly oxygenated red blood cells will augment the properties of blood cardioplegia, decreasing the incidence of PCXD and increasing survival. Further investigation of albumin and dextran containing cardioplegia with fresh red blood cells without NICP is currently underway to determine its efficacy compared with NICP. This may demonstrate a favorable alternative to NICP in cardiac xenotransplantation to reduce the incidence of PCXD and increase survival that is of decreased cost and complexity. That being said, blood cardioplegia must demonstrate non-inferiority in larger studies either alone, or in combination with other additives prior to replacing NICP as a durable alternative in cardiac xenotransplantation.

## Data Availability Statement

The raw data supporting the conclusions of this article will be made available by the authors, without undue reservation.

## Ethics Statement

The animal study was reviewed and approved by Institutional Animal Care and Use Committee (IACUC) at the University of Maryland School of Medicine.

## Author Contributions

CG, BG, AS, DK, and MM wrote, critique, edited the manuscript and conducted experiments. MA, SS, IK, BL, IT, AH, TZ, ES, PO, BW, AT, AB, and DA critique, edited the manuscript and conducted experiments. All authors contributed to the article and approved the submitted version.

## Funding

Funding of this study is generously provided by public funding-NIH U19 AI090959 “Genetically-engineered Pig Organ Transplantation into Non-Human Primates” and private funding by United Therapeutics. United Therapeutics was not involved in the study design, collection, analysis, interpretation of data, the writing of this article or the decision to submit it for publication.

## Conflict of Interest

DA is employed by Revivicor, Inc., a subsidiary of United Therapeutics.

The remaining authors declare that the research was conducted in the absence of any commercial or financial relationships that could be construed as a potential conflict of interest.
